# Full-length transcriptome analysis reveals the mechanism of acupuncture at PC6 improves cardiac function in myocardial ischemia model

**DOI:** 10.1186/s13020-021-00465-8

**Published:** 2021-07-08

**Authors:** Jing Yuan, Jun-Meng Wang, Zhi-Wei Li, Cheng-Shun Zhang, Bin Cheng, Su-Hao Yang, Bai-Tong Liu, Li-Juan Zhu, Ding-Jun Cai, Shu-Guang Yu

**Affiliations:** Acupuncture and Tuina School/Third Teaching Hospital, Chengdu University of Traditional Chinese Medicine, Chengdu, 610075 Sichuan Province China

**Keywords:** Acupuncture, Myocardial ischemia, Full-length transcriptome, Differential expression genes, Alternative splicing, New genes

## Abstract

**Background:**

The pathological process of myocardial ischemia (MI) is very complicated. Acupuncture at PC6 has been proved to be effective against MI injury, but the mechanism remains unclear. This study investigated the mechanism that underlies the effect of acupuncture on MI through full-length transcriptome.

**Methods:**

Adult male C57/BL6 mice were randomly divided into control, MI, and PC6 groups. Mice in MI and PC6 group generated MI model by ligating the left anterior descending (LAD) coronary artery. The samples were collected 5 days after acupuncture treatment.

**Results:**

The results showed that treatment by acupuncture improved cardiac function, decreased myocardial infraction area, and reduced the levels of cTnT and cTnI. Based on full-length transcriptome sequencing, 5083 differential expression genes (DEGs) and 324 DEGs were identified in the MI group and PC6 group, respectively. These genes regulated by acupuncture were mainly enriched in the inflammatory response pathway. Alternative splicing (AS) is a post-transcriptional action that contributes to the diversity of protein. In all samples, 8237 AS events associated with 1994 genes were found. Some differential AS-involved genes were enriched in the pathway related to heart disease. We also identified 602 new genes, 4 of which may the novel targets of acupuncture in MI.

**Conclusions:**

Our findings suggest that the effect of acupuncture on MI may be based on the multi-level regulation of the transcriptome.

**Supplementary Information:**

The online version contains supplementary material available at 10.1186/s13020-021-00465-8.

## Background

Myocardial ischemia (MI) is a severe cardiovascular disease that is the leading cause of morbidity and mortality [7]. Since the pathological process and mechanism of MI injury is complicated, which involved in cardiac fibrosis [29], inflammation [42], angiogenesis [28], apoptosis [2], the regulation of this disease is also multifaceted. As a complementary and alternative medical approach, acupuncture has been widely used to relieve the symptoms of angina and palpitations [24, 45]. Many studies have been proved the protective effect of acupuncture on MI [8, 17]. However, because of the extensive regulation effect of Acupuncture on various pathological processes of MI injury, the entire mechanism is also unclear.

Gene regulation is a fundamental process in developing and disease progression, and transcriptome research a necessary tool for understanding the gene expression profile. Therefore, we could like to research the mechanism of acupuncture's effect on MI from the transcriptome. The regulation of acupuncture on ischemia–reperfusion has been proved by some researches in transcriptome analysis [17]. However, the effect of acupuncture on genome-wide gene expressions in myocardial ischemia models has not been reported. Moreover, most researches on the transcriptome of acupuncture effect on heart diseases are relatively limited and primarily focuses on differential genes. In this study, full-length transcriptome sequencing was used to obtain a more comprehensive transcriptome profile to analyze the pathogenesis of MI and the regulatory effect of acupuncture from a more perspective. In fact, the transcriptome is complex, such as alternative splicing, LncRNA, and polyadenylation, which can also affect gene function [33, 46]. As a primary mechanism for producing protein diversity, alternative splicing has been demonstrated to be involved in proliferation, apoptosis, angiogenesis, and so on [44]. Alternative splicing also plays an essential role in heart disease [9, 25]. Research proved that alternative splicing in titin (TTN) could modify sarcomeric stiffness to influence cardiac function [34]. Mutations in a splicing factor of TTN, RBM20, have been proved involved in dilated cardiomyopathy [15]. However, the role of alternative splicing in myocardial ischemia has not been well understood. Based on full-length transcriptome, alternative splicing can be detected, which allows us to study the role of alternative splicing in MI. Moreover, some new genes which structure and function have not been reported can also be identified by full-length transcriptome, these new genes may be the critical targets for acupuncture.

To investigated the mechanism of acupuncture against MI injury from a more comprehensive perspective, we studied the transcriptome from the aspects of differential genes, alternative splicing, and new genes. This study aimed to investigate the mechanism of acupuncture against MI from the multi-level transcriptome. This research will help find new therapeutic targets for MI and a unique perspective for acupuncture research.

## Methods

### Experimental animals and grouping

Six to 8-week-old SPF (specific pathogen-free) C57BL/6 male mice (20–23 g) were purchased from Byrness Weil biotech Ltd (Hunan, China) and used in the experiments. Animals were fed adaptively for one week in the environment with a temperature of 23–25 °C and relative humidity of 45–55%. The study was performed according to the guidelines of the National Institutes of Health Animal Care and Use of Laboratory Animals (Bethesda, MD, USA). All the mice were divided into the Control group, MI group, and PC6 group randomly.

### Induction of MI in mice

The MI models were produced by ligating the left anterior descending coronary artery (LAD). Mice were anesthetized by inhalation of 5% isoflurane and maintained with 2% isoflurane (RWD, Shenzhen, China). The skin was incised along the left side of the sternum, and the muscles were bluntly separated, then incised between the 3rd and 4th intercostal spaces and permanent LAD ligation. In the control group, the same procedure was performed except for the LAD ligation. The Lead II Electrocardiogram was monitored before and after the operation.

### Acupuncture intervention

The PC6 group mice underwent acupuncture treatment after 24 h of MI operation bilateral PC6 acupoint once a day under awake, for a total of 5 days. Fold the acupuncture needles into an “L” shape, then inserted needles into PC6 acupoint about 1–2 mm. After the needles were fixed with adhesive tape, mice were put into the cages and moved freely. Then we pressed the needles every 5 min to ensure the needles would not fall off and strengthen the stimulation of the acupuncture intervention. The needles were removed after 20 min of treatment.

### Electrocardiogram (ECG) and echocardiography

Before and after the MI operation, the electrocardiogram (lead II) of each group of mice was recorded by the Power Lab system (AD Instruments, Australia). Then, 5 min after LAD surgery, the ST-segment was elevated to the myocardial ischemic indication. 5-day after MI, the cardiac function was assessed by transthoracic echocardiography (Beijing Yeeran Technology Co., Ltd). Mice have maintained anesthesia with 1.5% isoflurane, and heart rate was maintained at about 450–550 bpm during the examination. The indices included left ventricular end-diastolic diameter (LVEDD) and left ventricular end-systolic diameter (LVESD). The left ventricular ejection fraction (LVEF) and left ventricular fractional shortening (LVFS) were calculated according to LVEDD and LVESD. The LVEF and LVFS were calculated as follows: EF = [(LV Vol: d-LV Vol: s)/LV Vol: d] × 100%; FS = [(LVEDDLVESD)/LVEDD] × 100%.

### Triphenyitertrazolium chloride (TTC) staining

TTC staining was used to assess the infarct area in heart tissue. Mice were sacrificed five days after acupuncture treatment, and the heart tissues were frozen for 20 min at – 20 °C and cut into five pieces below the ligature. The slices were stained in 2% TTC solution (Sigma Chemical Co., USA), incubated at 37 °C for 20 min and fixed in 4% paraformaldehyde for 4 h. The infarct area was assessed by the use of Image-Pro Plus 6.0.

### Collection of tissue and serum samples

After 5 days of acupuncture treatment, the mice were sacrificed under deep anesthesia with pentobarbital sodium (100 mg/kg) administered by intraperitoneal injection. Blood was collected from the orbital vein and then centrifuged for 10 min at 4000 rpm, 4 °C, to obtain the serum. The serums were stored at − 80 °C for myocardial enzymes and inflammatory cytokines determination.

### Determination of serum myocardial enzymes and inflammatory cytokines

The levels of cTnT, cTnI, TNF-α, IL-1β, and IL-6 were quantified with the ELISA kits according to the manufacture’s protocol (E-EL-M1801c, E-EL-M1203c, E-EL-M0049c, E-EL-M0037c and E-EL-M2453c, Elabscience, China).

### RNA extraction, library construction and sequencing

Total RNA was extracted from 9 heart samples and prepared for cDNA libraries using the protocol provided by Oxford Nanopore Technologies (ONT). Briefly, SuperScript IV First-Strand Synthesis System (Invitrogen) was used for full-length mRNA reverse transcription and following cDNA PCR for 14 circles with LongAmp Tag (NEB). The PCR products were then subjected to FFPE DNA repair and end-repair (NEB) steps and following adaptor ligation using T4 DNA ligase (NEB). Agencourt XP beads were used for DNA purification according to ONT protocol. The final cDNA libraries were added to FLO-MIN109 flowcells and were run on the PromethION platform at Biomarker Technology Company (Beijing, China).

### Analysis for RNA-seq data

Raw reads were first filtered with minimum average read quality score = 7 and minimum read length = 500 bp. Ribosomal RNA was discarded after mapping to the rRNA database. Next, full-length, non-chimeric (FLNC) transcripts were determined by searching for primer at both ends of reads. Clusters of FLNC transcripts were obtained after mapping to the reference genome with mimimap2, and consensus isoforms were obtained after polishing within each cluster by pinfish. Transcripts were validated against known reference transcript annotations with gffcompare. AS events including IR, ES, AD, AA, and MEE were identified by the AStalavista tool. Differential expression analysis of two groups was performed using the DESeq R package (1.18.0). DESeq provides statistical routines for determining differential expression in digital gene expression data using a model based on the negative binomial distribution. The resulting P values were adjusted using Benjamini and Hochberg’s approach for controlling the false discovery rate. Genes with a *P* value < 0.05 and fold change ≥ 1.5 found by DESeq were assigned as differentially expressed. Gene Ontology (GO) enrichment analysis of the differentially expressed genes (DEGs) was implemented by the GOseq R packages based on Wallenius non-central hypergeometric distribution, which can adjust for gene length bias in DEGs. KEGG analysis was used KOBAS software to test the statistical enrichment of differential expression genes in KEGG pathways.

### Hematoxylin–eosin (HE) staining

The heart tissues in the control, MI, and PC6 group were collected and fixed with buffered 4% formalin for two days. All the samples were sectioned at a thickness of 5 µm and stained with hematoxylin and eosin for histopathologic observation by light microscopy (Olympus Corporation, Japan).

### Real-time fluorescence quantitative polymerase chain reaction (RT-qPCR)

Total mRNA was isolated from the heart tissues using MolPure^®^ TRIeasy™ Plus Total RNA Kit (YEASEN, Shanghai, China), and cDNA was prepared using Hifair^®^ III 1st Strand cDNA Synthesis SuperMix for qPCR (YEASEN, Shanghai, China) according to the manufacturer’s instructions. The mRNA levels were assessed on a bio-rad cfx maestro system Real-time PCR system (Bio-Rad, USA) by qPCR using Hieff^®^ qPCR SYBR^®^ Green Master Mix (YEASEN, Shanghai, China). The relative expression of mRNA was calculated by △△Ct according to standard methods. The primer sequences as follows:

Cxcl2, forward: 5′-CCAACCACCAGGCTACAGG-3′, reverse: 5′-GCGTCACACTCAAGCTCTG-3′; Saa3, forward: 5′-TGCCATCATTCTTTGCATCTTGA-3′, reverse: 5′-CCGTGAACTTCTGAACAGCCT-3′; Smad1, forward: 5′-GGGGGATCCGTAATGTGACAAGTTTATTTTC-3′, reverse: 5′-TTTGCGGCCGCTCAAGATACAGATGAAATAG-3′; Arg1, forward: 5′-AGCTCTGGGAATCTGCATGG-3′, reverse: 5′-ATGTACACGATGTCTTTGGCAGATA-3′; GAPDH, forward: 5′-TGTGTCCGTCGTGGATCTGA-3′, reverse: 5′-CCTGCTTCACCACCTTCTTGA-3′.

### Statistical analysis

Statistical analysis was completed using SPSS 19.0 software (IBM, Chicago, USA). Values are presented as mean ± SEM. The two-group comparison was determined using the unpaired Student’s t-test, and one-way ANOVA made multiple-group comparisons. *P* < 0.05 was considered statistically significant.

## Results

### Acupuncture treatment at the PC6 acupoint effectively protected the myocardium from MI injury

**Fig. 1:**
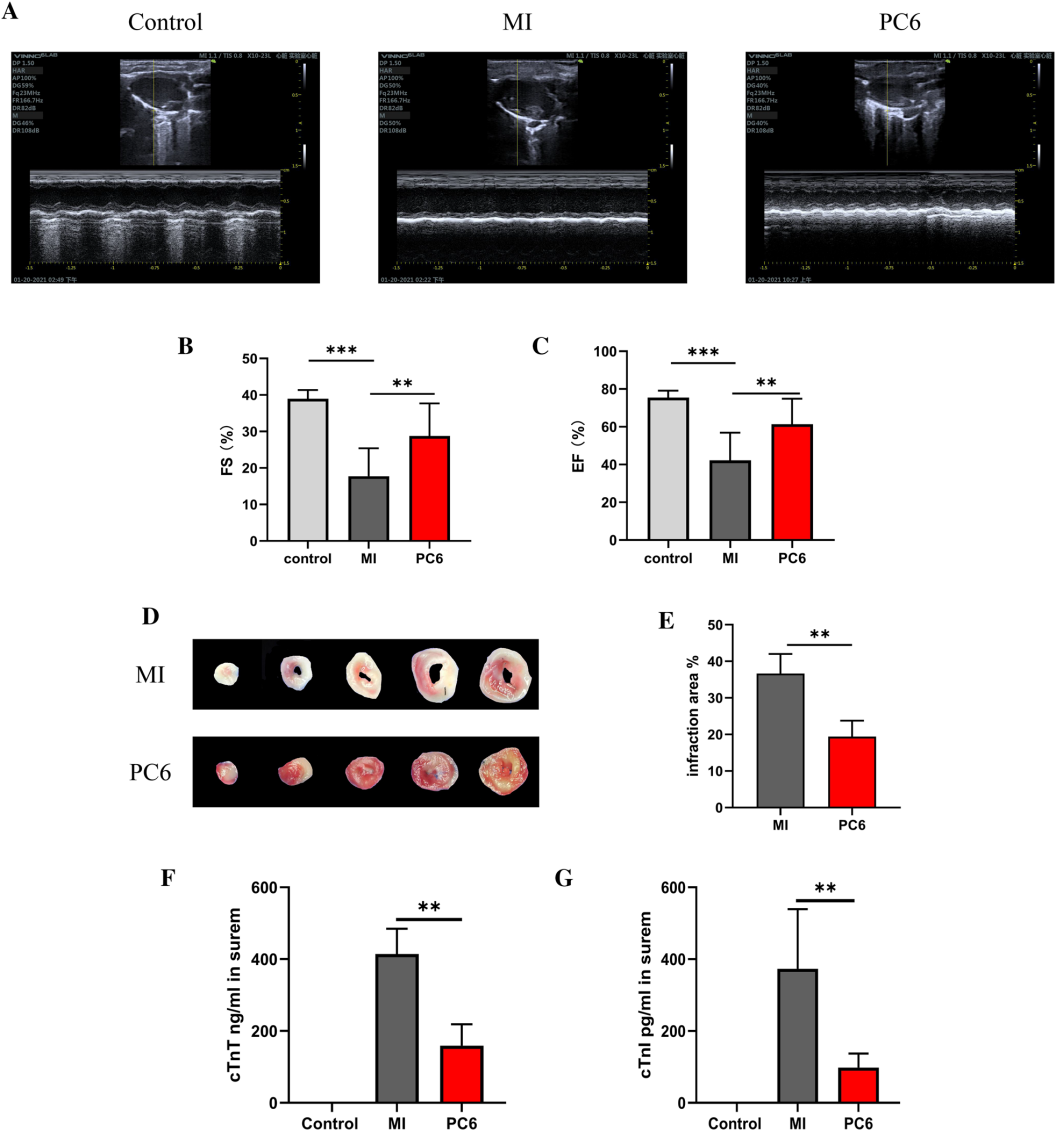
Acupuncture at PC6 alleviates myocardial ischemia injury. **A** Representative echocardiographic images of Control, MI, and PC6 group. **B**, **C** Statistics of echocardiography include FS (**B**) and EF (**C**) of each group of mice. **D** Representative TTC staining of hearts from MI and PC6 group. **E** Myocardial infarction injury size percentage of MI and PC6 group. **F**, **G** The cTnT (**F**) and cTnI (**G**) levels in the serum of mice in each group. **P < 0.01; ***P < 0.001, n = 6

To investigate the effects of acupuncture at PC6 acupoint on myocardial injury, we first assessed the cardiac function and infarct area. Echocardiography results indicated that both EF and FS significantly decreased after MI compared with the control group. After treatment at PC6 acupoint, both EF and FS increased compared to the MI group (Fig. 1A–C). The infarct area was assessed at 5d after acupuncture treatment by TTC staining. The results showed that acupuncture treatment significantly decreases the myocardial injury size (Fig. 1D, E). The levels of myocardial-specific serum enzymes, including cardiac troponin T (cTnT) and cardiac troponin I (cTnI), which reflect acute myocardial injury, were measured by ELISA. The results indicated that the levels of cTnT and cTnI were increased after MI operation, acupuncture significantly decreased the levels of serum enzymes (Fig. 1F, G).

### Acupuncture at the PC6 acupoint altered genes expressions genome-widely

**Fig. 2:**
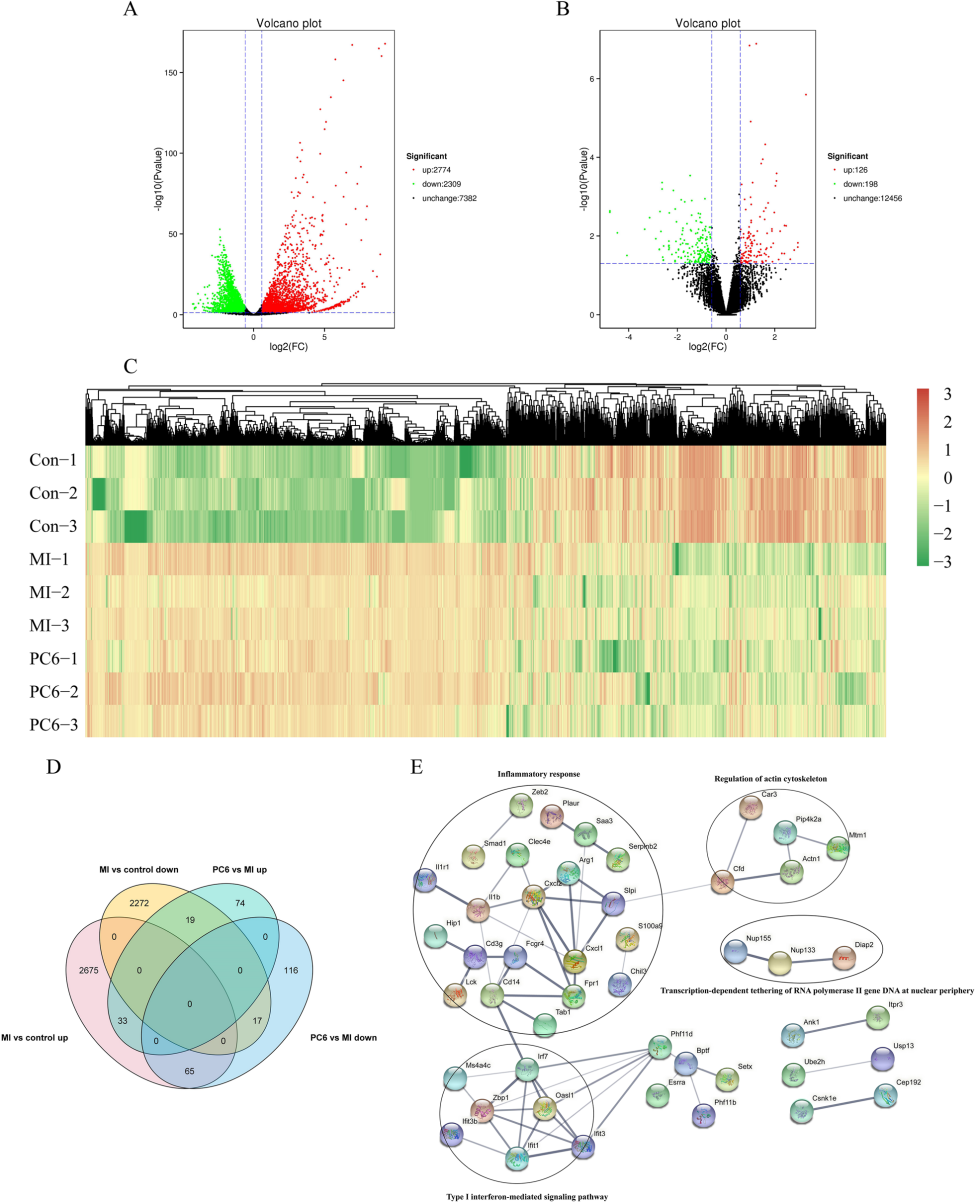
DEGs analysis identified by Full-length transcriptome. **A**, **B** Volcano Plot for DEGs in MI group compared to control group (**A**), and DEGs in PC6 group compared to MI group (**B**). **C** The heatmap of up-regulated genes and down-regulated genes in the control group, MI group, and PC6 group, respectively. **D** Venn Diagram of DEGs. **E** PPI network of DEGs, which co-regulated by MI and PC6. *p* < 0.05, n = 3

To explore the mechanism of acupuncture treatment on MI, we completed gene expression profiling of different groups using Nanopore. RNA-seq results showed that compared to the control group, 5083 genes were differentially expressed in the MI group; of these, 2774 genes were up-regulated, and 2309 genes were down-regulated (Fig. 2A; Additional file 1: Table S1). We also found 324 differentially expressed genes (DEGs) in the PC6 group compared to the MI group; 126 differentially expressed genes were up-regulated, and 198 genes were down-regulated (Fig. 2B; Additional file 2: Table S2).

We further analyzed the gene expression patterns by clustering ONT data. The heatmaps were created with the up-regulated genes and down-regulated genes in the control group, MI group, and PC6 group. As Fig. 2C showed, the red color was higher, and the green color illustrated lower expression genes. Then, we analyze overlaps of the differential genes with Venn Diagrams (Fig. 2D). In the 2773 up-regulated DEGs of the MI group, 65 DEGs were down-regulated by acupuncture treatment. On the other side, in the 2308 down-regulated DEGs of the MI group, 19 DEGs were reversed by acupuncture treatment. We speculate that these 84 DEGs may be the key targets of the acupuncture effect against myocardial ischemia. Additionally, the DEGs co-regulated by MI and PC6 were subjected to protein–protein interaction (PPI) network analysis based on the STRING database (Fig. 2E). And it showed that proteins were clustered into four categories: inflammatory response, Regulation of actin cytoskeleton, Type I interferon-mediated signaling pathway, and Transcription-dependent tethering of RNA polymerase II gene DNA at the nuclear periphery. Our data set offered an insight into the interactions of proteins in the MI pathological processes and provided a guidance for the target genes of acupuncture.

### The validations of inflammatory response

**Fig. 3:**
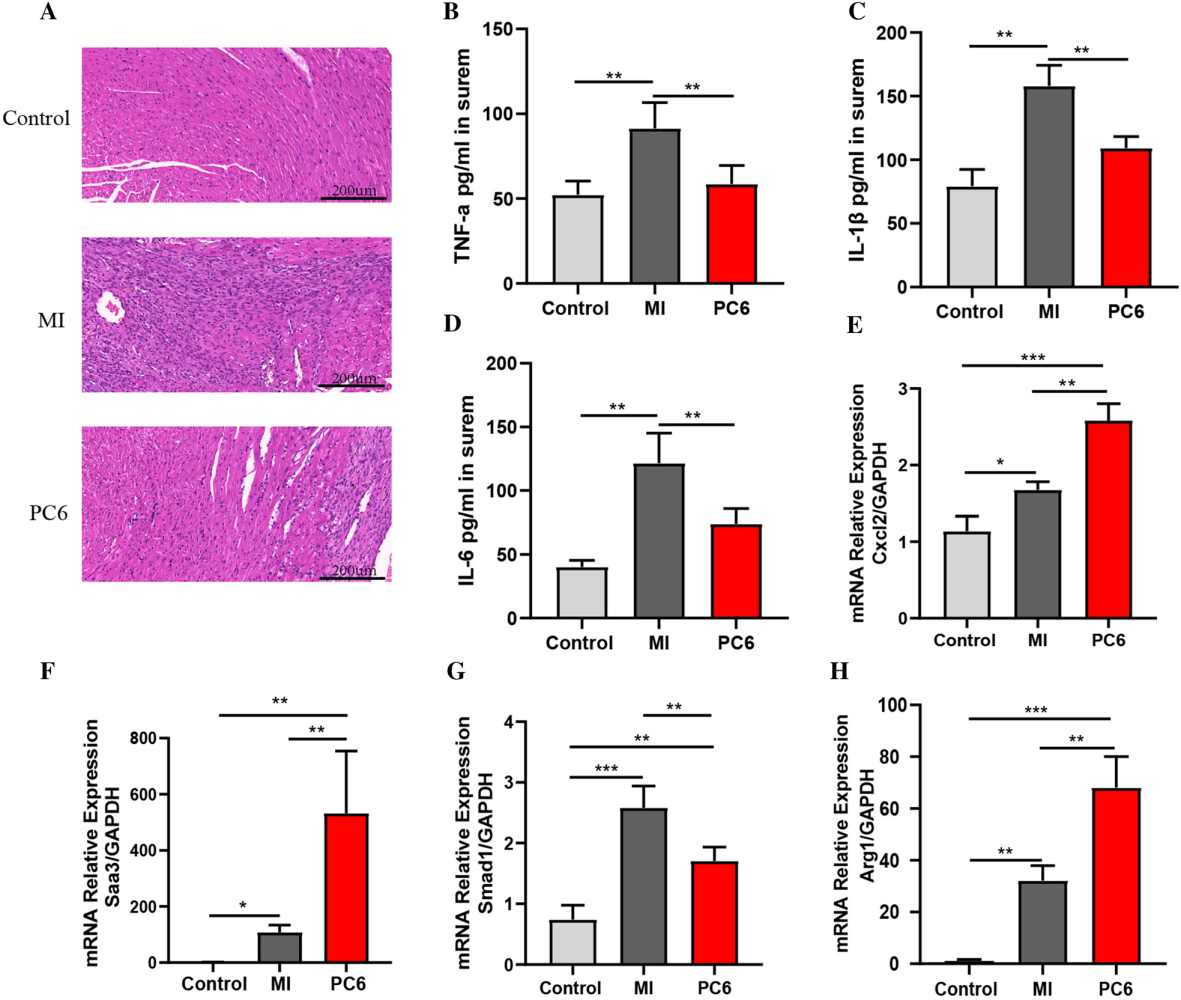
Verification of inflammatory response. **A** HE staining of heart tissue in each group (n = 4). **B**–**D** The levels of TNF-α (**F**), IL-1β (**G**), and IL-6 (**H**) in the serum of mice in each group (n = 6). **E** Expression of CXC motif chemokine ligand 2 (Cxcl2). **F** Expression of serum amyloid antigen 3 (Saa3). **G** Expression of mothers against decapentaplegic homolog 1 (Smad1). **H** Expression of Arginase-1 (Arg1) (n = 4). *P < 0.05, **P < 0.01, ***P < 0.001. HE staining was magnified by 200 ×

We found that most of the DEGs are primarily enriched in the inflammatory response signaling pathway so that we observed inflammation by HE staining, Elisa and qPCR. HE staining showed a massive accumulation of infiltrated cells in the MI group, and the inflammatory cell infiltration was significantly decreased by acupuncture (Fig. 3A). We also detected the levels of inflammatory cytokines in serum. As revealed in Fig. 3B–D, the levels of TNF-α (tumor necrosis factor-α), IL-1β (interleukin-1β), and IL-6 (interleukin-6) in serum were increased in the MI group compared with the control group, and the levels of these inflammatory cytokines were decreased by acupuncture. Then, we verified some of the DEGs, including Cxcl2, Saa3, Smad1, and Arg1, which involved inflammatory response by qPCR. The qPCR confirmed a similar tendency as shown in RNA-seq data in Additional files 1, 2: Tables S1, S2 (Fig. 3E–H). These results proved the RNA-seq data was reliable and confirmed the regulation effect of acupuncture on inflammation.

### Integrating alternative splicing events in myocardial ischemia

**Fig. 4:**
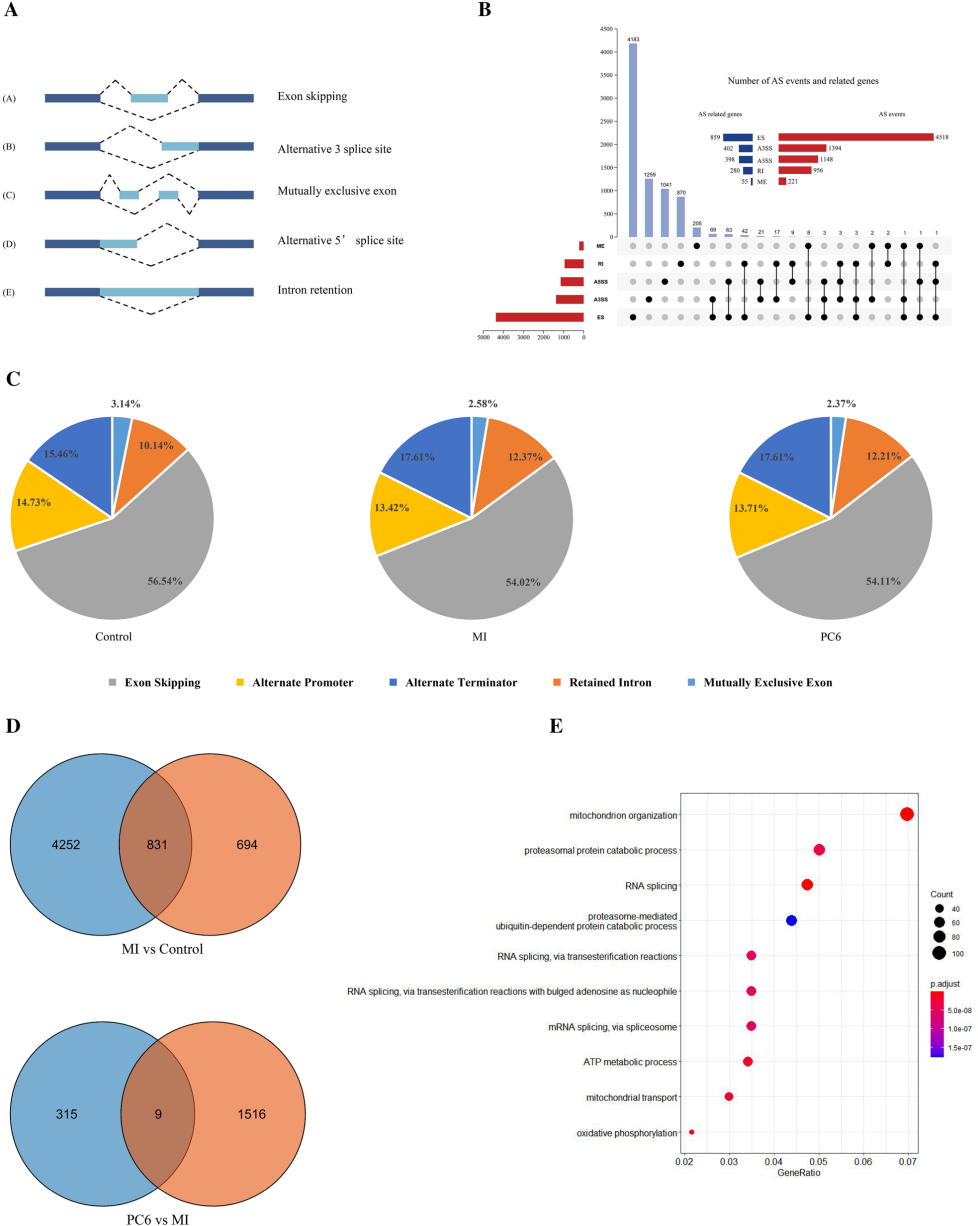
Overview of AS events in MI. **A** Typical patterns of AS events. **B** UpSet plot of AS events and their interactions between genes. One gene may have several types of AS events. **C** Proportions of AS events in each group. **D** Venn Diagrams of DEGs and AS events involved genes. **E** KEGG pathway enrichment of AS events involved genes

Alternative splicing (AS) is one of the primary mechanisms for producing protein diversity. Previous researches have reported that AS is involved in the development of myocardial ischemia [35]. We also identified a large number of AS in each group. There are five main types of AS, including Exon skipping (ES), Intron retention (IR), Alternative 3′ splice site (A3SS), Alternative 5′ splice site (A5SS), and Mutually exclusive exon (ME) (Fig. 4A). In all samples detected by RNA-seq, 8237 AS events associated with 1994 genes were found. We detected 4518 ES-types AS events involving 859 genes, 1394 A3SS-type AS events containing 402 genes, 1148 A5SS-type AS events involving 398 genes, 956 RI-types AS events involving 280 genes, 221 ME-types AS events involving 55 genes, as shown in Fig. 4B. The results indicated that one gene might have several types of mRNA splicing events; some genes may be expressed by 3 AS types. The proportion of the five types of AS were shown in Fig. 4C. We found that more than half of the AS events were ES events. Then we analyzed differentially expressed genes and AS events involved genes (Fig. 4D). The results showed many DEGs underwent AS events, indicating that many genes may play a role in the pathological process of MI and acupuncture treatment through alternative splicing. Kyoto Encyclopedia of Genes and Genomes (KEGG) pathway enrichment of AS involved genes found that the pathways including mitochondrion organization, proteasomal protein catabolic process, RNA splicing, mRNA splicing via spliceosome, and metabolic process were the central pathways in which the AS involved genes enriched (Fig. 4E).

### The analysis of differential alternative splicing events in myocardial ischemia

**Fig. 5:**
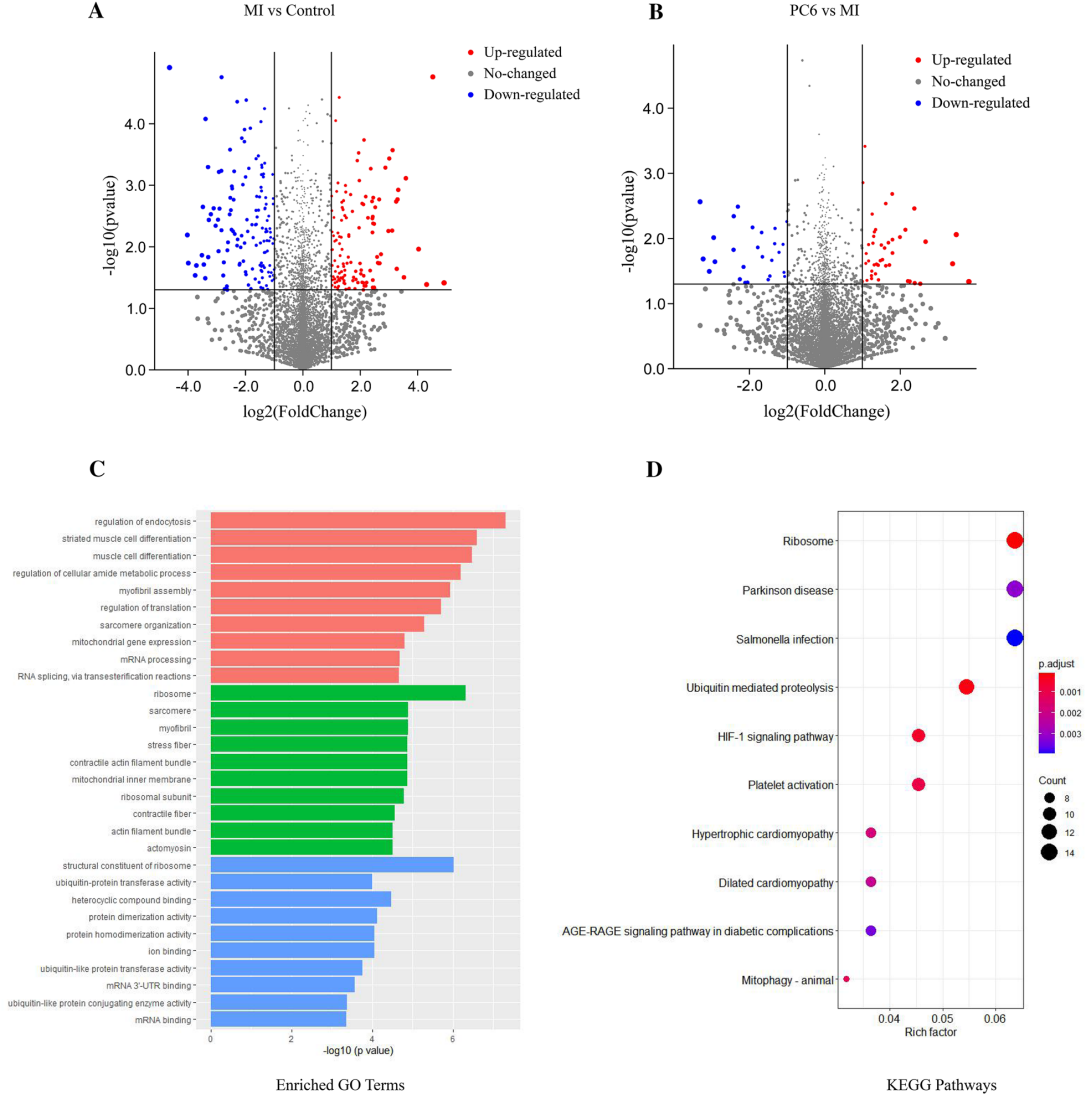
The analysis of differential alternative splicing events. **A**, **B** Volcano plot of differential AS events in MI group compared to control group (**A**) and in PC6 group compared to MI group (**B**). **C** GO terms of differential AS events involved genes. **D** KEGG pathways of differential AS events involved genes

Differential AS analysis revealed that 347 differential AS events were identified in this research, of which 174 were differentially upregulated AS events, 173 were differentially downregulated AS events, the numbers of upregulated AS events were equal to downregulated AS events (Fig. 5A, B). There were 129 differentially upregulated AS events and 147 differentially downregulated AS events in the MI group compared to the control group. There were 45 differentially upregulated AS events and 26 differentially downregulated AS events in the PC6 group compared to the MI group (Fig. 5A, B). To further explore the genes involved in differential AS events, GO annotations and KEGG pathways were used. These genes were annotated through three independent ontologies in the GO database, including biological process (BP), molecular function (MF), and the cellular component (CC). The top 10 GO terms of BP, MF, and CC were showed in Fig. 5C. Through the KEGG enrichment analysis, we found that the top 10 pathways which these genes enriched including Ribosome, HIF-1 signaling pathway, Hypertrophic cardiomyopathy, Dilated cardiomyopathy (Fig. 5D). The results suggested that these genes involved in differential AS events were associated with heart disease.

### The analysis of new genes detected in full-length transcriptome

**Fig. 6:**
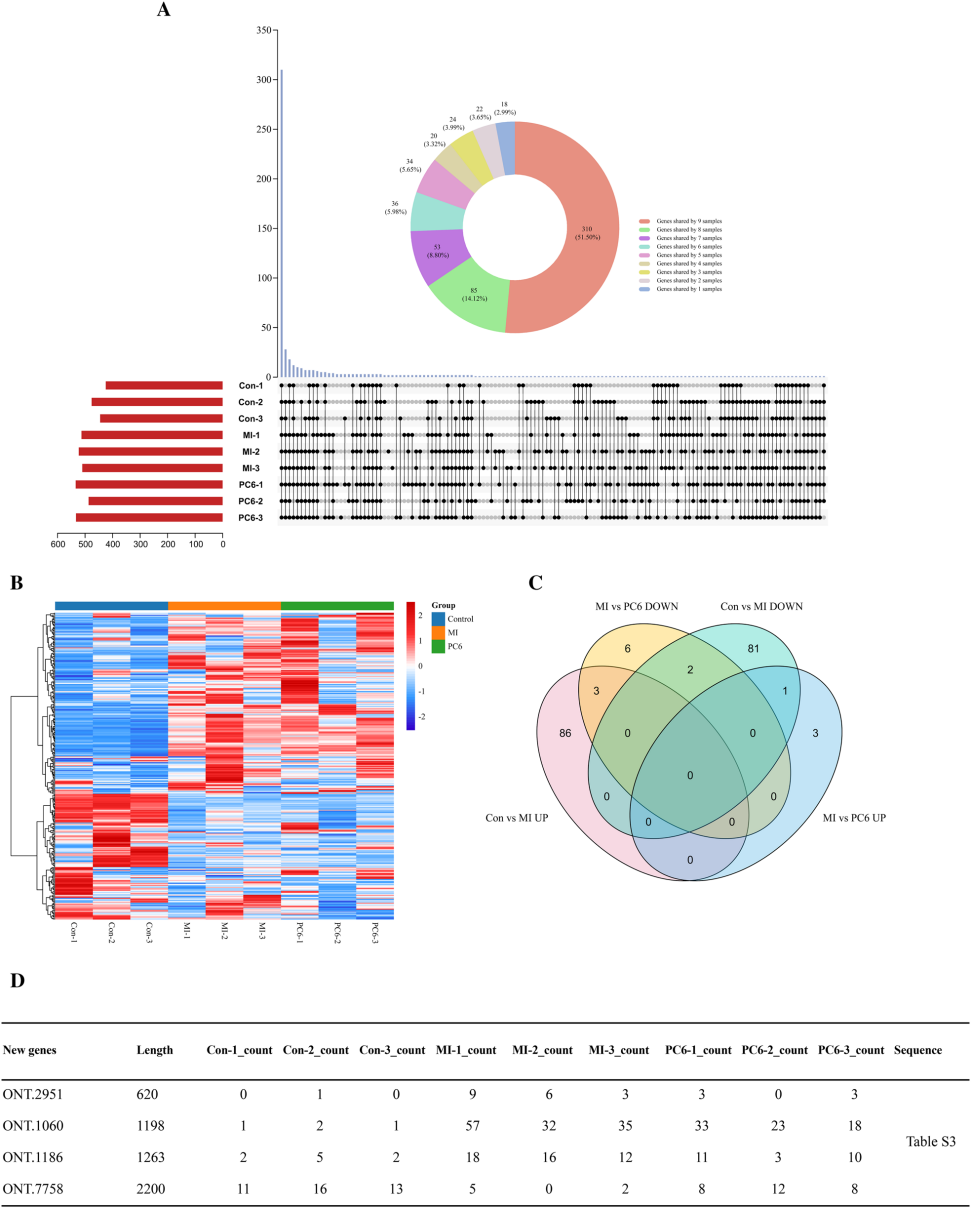
The analysis of new genes. **A** UpSet plot of new genes in 9 samples. **B** The heatmap of new genes in the control group, MI group, and PC6 group. **C** Venn Diagram of differential new genes in MI group compared to control group and in PC6 group compared to MI group. **D** The expression of differential new genes in each sample

Full-length sequencing can accurately identify the structure of transcripts. Gffcompare is used to align transcripts obtained by full-length sequencing to known transcripts of the genome, so novel genes and transcripts can be obtained to supplement genome annotation. In this study, we found 602 new genes. Most of these new genes were shared by all 9 samples, which suggested that these new genes can be stably expressed in all samples (Fig. 6A). We also noticed that some new genes were expressed only in the MI group and PC6 group, indicated the gene expression pattern was changed under the pathological condition (Fig. 6A). The heatmap was created with all the new genes of each group. As Fig. 6B showed, the new gene expression patterns of control were significantly different with the MI group and PC6 group; however, the MI group and PC6 group had similar new gene expression patterns. We analyzed overlaps of differential new genes with Venn Diagrams. And found that 3 up-regulated new genes in the MI group were down-regulated by acupuncture; only 1 down-regulated new gene in the MI group was up-regulated by acupuncture (Fig. 6C). The expression and sequence of these 4 new genes were showed in Fig. 5D and Additional file 3: Table S3.

## Discussion

Myocardial ischemia is the leading cause of disability and death worldwide [16]. As a traditional Chinese treatment, acupuncture has been proven to have a protective effect against myocardial ischemic injury [38, 39, 41]. Previous researches have indicated that acupuncture at PC6 can attenuate ischemia injury by reducing apoptosis [37], correcting arrhythmia [10], improving energy metabolism [41]. Our results found that acupuncture at PC6 could protect the heart from myocardial ischemia injury. In this study, MI models were performed by ligating the LAD, which was a classic mouse MI model [22]. And then applied acupuncture intervention to these MI models. We evaluated myocardial injury by using ECG, echocardiography, TTC staining, and myocardial enzyme levels. Echocardiographic EF and FS mainly estimated systolic cardiac function [31]; these results showed that both EF and FS were significantly decreased in the MI group. TTC staining showed a significant myocardial infraction and high levels of cTnT and cTnI in MI models. Acupuncture at PC6 not only increased EF and FS, and decreased the injury area and the levels of the myocardial enzyme. These findings were consistent with the previous research [8, 17, 42].

MI dramatically modified the gene expression pattern of the tissue environment, and gene expression is fundamental and essential in disease progression and treatment. Therefore, transcriptome research is one of the necessary tools for understanding life processes. To investigated the mechanism of acupuncture against MI injury from a more comprehensive perspective, we studied the transcriptome from the aspects of differential genes, alternative splicing, and new genes by full-length transcriptome. In previous studies on MI, Second-generation sequencing technology (SGS) has been widely used [11, 23, 40, 48]. However, the short-read RNA-seq method of SGS is limited to identify and quantify complex transcript isoforms. Identification of all transcripts produced by a gene is essential to research the disease progression. Thus, the ability to produce longer reads would be a better choice to understand transcriptome complexity, including AS, polyadenylation, and fusion genes [1, 30, 43]. This study applied the third-generation sequencing technology (TGS), commercialized by Oxford Nanopore Technologies (ONT) MinION, to research the transcriptome complexity in MI and acupuncture treatment. Compared with SGS, the read length of TGS can be about 1.5 kbp, and the transcriptome information is more comprehensive.

In this study, we found that the gene expression patterns were significantly changed after MI, acupuncture also could regulate some of these genes. By analyzing differentially expressed genes in MI and PC6 group, we found 5083 DEGs (2774 up-regulated and 2309 down-regulated) in the MI group compared to the control group; 324 DEGs (126 up-regulated and 198 down-regulated) in the PC6 group compared to the MI group. The analysis of DEGs, which co-regulated in two groups, indicated that these genes were mainly enriched in Inflammatory response, Regulation of actin cytoskeleton, Type I interferon-mediated signaling pathway, and Transcription-dependent tethering of RNA polymerase II gene DNA at the nuclear periphery. The effects of acupuncture on some of these signaling pathways have been reported in previous research [36, 42], suggesting that these signaling may be the key targets of acupuncture. Since most of these co-regulated genes are involved in the inflammatory response, and the inflammatory response plays a crucial role in scar formation and myocardial remodeling in MI [6] so that we verified the inflammatory reaction in MI and the regulatory effect of acupuncture on it from the morphological, gene and protein levels. We firstly verified the DEGs involved in the inflammatory response by qPCR. These genes, including Cxcl2, Arg1, and Saa3 play a key role in inflammatory response [13, 18, 21]. The role of Cxcl2 and Smad1 in myocardial ischemia also have been reported [12, 26, 32]. We found that qPCR results were consistent with RNA-seq, indicating that our RNA-seq data are reliable. HE staining showed that the inflammatory infiltration of the ischemic myocardium was reduced by acupuncture. TNF-α, IL-1β, and IL-6 are the major pro-inflammatory cytokines that aggravate the inflammatory response after ischemia injury. Our results also confirmed that acupuncture could decrease the level of these inflammatory cytokines in serum, further proving the anti-inflammatory effect of acupuncture.

In recent years, alternative splicing, another factor that causes protein diversity, has also been widely studied in many fields [4, 27, 33]. The pre-mRNA generated by gene transcription has many splicing methods. Different exons are selected to produce different mature mRNAs, then they are translated into different proteins, and these proteins can bring diversity to biological traits [5, 47]. The process of pre-mRNA treatment is called alternative splicing. There are five main types of AS, including ES, RI, ME, A3SS, A5SS, and ES, the most common AS in mammals. In recent years, there are many studies on AS in the cardiovascular field. In the heart, alternative splicing of sarcomeric genes, ion channels, and cell signaling proteins can cause cardiomyopathies and arrhythmias [19, 20]. Research on cardiovascular disease found that abnormalities in alternative splicing of apoptotic genes, including the Bcl-2 family, Caspases, Binp3, and Nix, are associated with many heart diseases [4]. Alternative splicing disorder of CaMKIIδ can cause LDH release, ATP reduction, and ROS accumulation to aggravate hypoxia-reoxygenation injury of cardiomyocytes [25]. Alternative splicing factor-regulated alternative splicing of CaMKIIδ also plays a vital role in pathological cardiac remodeling in heart failure [14]. Although alternative splicing is essential in heart disease research, its role in myocardial ischemia is still not well understood, and the effect of acupuncture on alternative splicing has not been reported. This study first analyzed AS events involved genes and found that these genes were mainly enriched in pathways associated with RNA splicing and energy metabolism. Moreover, a considerable number of AS events involved genes showed no difference in expression through the analysis of overlap. These genes enhanced the diversity of protein by AS events but do not affect gene expression. Analysis of differential alternative splicing found that DAS involved genes were enriched in signaling pathways associated with heart disease, including the hypoxia-inducible transcription factor-1 (HIF-1) signaling pathway, Hypertrophic cardiomyopathy, Dilated cardiomyopathy. These results indicated that the regulation of AS events might also be a possible target of acupuncture. In addition, HIF-1 is also a global regulator of macrophage and neutrophil inflammatory [49. It also have been reported to play a role in inflammatory response after MI by interacting with Pyruvate kinase isozyme type M2 (PKM2) to influence macrophage M2 polarization [3]. This finding is consistent with the regulation of inflammation by DEGs. Since DEGs and alternative splicing both have a relationship with inflammation after MI, the inflammatory response may be a target to research the interaction between DEGs and alternative splicing.

Full-length sequencing can identify new genes. In this study, we found a total of 602 new genes detected in 9 samples sequenced. More than half of these new genes were expressed in all samples, suggest that these genes were relatively stable. Some genes were only expressed in the MI and/ or PC6 groups, indicating that these genes may be induced under the pathological condition. Finally, we detected 89 new genes were up-regulated in the MI group; 3 of these genes were down-regulated in the PC6 group; 83 new genes were down-regulated in the MI group, 1 of these genes was up-regulated in the PC6 group. The structure and function of these genes have not been reported in previous research, but the expression of these genes changes in MI condition and also can be regulated by acupuncture, we speculate that these 4 new genes may be a novel target of acupuncture.

This study provided a more comprehensive profile of the transcriptome regulated by acupuncture in MI models; however, in this study, only the DEGs were validated. Further research on critical genes regulated by alternative splicing and new genes is need.

## Conclusion

We demonstrate that acupuncture at PC6 could improve cardiac function and reduce infarct injury. By using Full-length transcriptome, we found that acupuncture can regulate differential expression genes and alternative splicing which may affect the structure and function of the protein. We also detected 4 new genes, which may be the target of acupuncture in the MI situation. Based on these results, we concluded that acupuncture might against myocardial ischemia through the regulation of multifaceted of the transcriptome.

## Supplementary Information

**Additional file 1: Table S1.** The differentially expressed genes in MI group compared to Control group.

**Additional file 2: Table S2.** The differentially expressed genes in PC6 group compared to MI group.

**Additional file 3: Table S3.** The sequences of new genes.

## Data Availability

The datasets used and/or analysed during the current study are available from the corresponding author on reasonable request.

## Abbreviations

MI: Myocardial ischemia
DEGs: Differential expression genes
cTnI: Cardiac troponin I
ECG: Electrocardiogram
LVEDD: Left ventricular end diastolic diameter
LVESD: Left ventricular end systolic diameter
ES: Exon skipping
A3SS: Alternative 3′ splice site
ME: Mutually exclusive exon
HE: Hematoxylin–eosin
Saa3: Serum amyloid antigen 3
Smad1: Mothers against decapentaplegic homolog 1
TNF-α: Tumor necrosis factor-α
LAD: Left anterior descending
AS: Alternative splicing
cTnT: Cardiac troponin T
LVEF: Left ventricular ejection fraction
LVFS: Left ventricular fractional shortening
TTC: Triphenyitertrazolium chloride
PPI: Protein–protein interaction
IR: Intron retention
A5SS: Alternative 5′ splice site
Cxcl2: CXC motif chemokine ligand 2
Arg1: Arginase-1
IL-1β: Interleukin-1β
IL-6: Interleukin-6
